# Comorbidities Burden and Implementation of the Treat–to–Target Strategy in Predicting Real-World Patient Outcomes in Spondyloarthritides

**DOI:** 10.31138/mjr.310723.cba

**Published:** 2023-07-31

**Authors:** Irini D. Flouri, Argyro Repa, Nestor Avgoustidis, Sofia Pitsigavdaki, Katerina Pateromichelaki, Eleni Marolachaki, Maria Terizaki, Myrto Nikoloudaki, Anastasios Eskitzis, Eleni Kalogiannaki, George Bertsias, Prodromos Sidiropoulos

**Affiliations:** Rheumatology and Clinical Immunology, University of Crete Medical School and University Hospital of Iraklio, Iraklio, Greece

**Keywords:** spondyloarthritis, psoriatic arthritis, treat-to-target, comorbidities, ankylosing spondylitis, compliance, predictors, registry

## Abstract

New biologic and small molecule targeted agents have expanded the armamentarium of Spondyloarthritides (SpA), allowing more therapeutic options for patients who do not respond to therapy. The implementation of the treat-to-target (T2T) strategy with close monitoring and frequent treatment adaptations targeting disease remission has been proposed as the means to prevent radiographic progression and long-term adverse outcomes. In this project we will employ the “University of Crete Rheumatology Clinic Registry” to prospectively study in real-world practice musculoskeletal and extraarticular disease activity, patient function, comorbidities, sociodemographics, imaging, compliance to therapy and other lifestyle factors in axial and peripheral SpA patients. The predictive value of these variables in long-term (2years) outcomes will be evaluated. We will also assess the implementation of the T2T approach as well as its impact on long-term patients’ outcomes (quality of life, productivity, adverse events). The successful completion of this study could pave the way for improved and personalized therapy in patients with SpA.

## BACKGROUND AND STUDY RATIONALE

Spondyloarthritides (SpA) are chronic diseases which cause inflammation at musculoskeletal as well as extraarticular sites, resulting in heterogeneous clinical phenotypes and variable organ damage. SpA are usually diagnosed in young to middle-aged adults and severely impact patients’ quality of life thereafter by the significant decline in physical function and their capacity to work. The chronic inflammatory state of SpA, as well as the cumulative drug exposure are also associated with a higher incidence of comorbidities in these patients, such as accelerated atherosclerosis with resultant cardiovascular disease, metabolic syndrome, osteoporosis with fractures and gastroduodenal ulcers.^1^ In addition, SpA are associated with a profound psychological burden, with high prevalence of depression and anxiety,^2^ as well as widespread musculoskeletal pain (fibromyalgia) caused by central pain sensitisation.^3^

Important steps to improve the management and outcomes of patients with SpA have been accomplished over the past decades, most notably by the development of validated, reliable metrics for the assessment of disease activity which directly relate to long-term outcomes and the recognition of the importance of early diagnosis and prompt initiation of effective therapy.^4^ Moreover, the concept of tight follow-up of patients with appropriate treatment adaptations in a treat-to-target (T2T) approach has also been introduced.^5,6^ In this regard, disease remission (or at least low disease activity) is the aim of therapy and the only means to prevent radiographic progression and long-term adverse outcomes.^7^

These steps were complemented by the breakthrough expansion of the therapeutic armamentarium for SpA, which now includes not only non-steroidal anti-inflammatory drugs (NSAIDs) and conventional synthetic disease modifying antirheumatic drugs (csDMARDs), but also biologic DMARDs (bDMARDs) and, recently, targeted synthetic DMARDs (tsDMARDs).^8,9^

Notwithstanding these important steps, many patients of everyday clinical practice do not achieve low disease activity (LDA), let alone remission, even with the newest bDMARD and tsDMARD therapies.^10^ In a recent analysis of SpA patients from the Hellenic Registry of Biologic Therapies (HeRBT) from 8 rheumatologic centres in Greece it was found that 58% of patients with axial disease and 80% of patients with peripheral joint involvement never achieve a state of inactive disease at any time point within the 1st year of TNFi therapy.^11^ In these patients it is not known if a T2T strategy was implemented as the T2T recommendations by the European Rheumatology Society (EULAR) were only recently issued.^12,13^ and the feasibility, as well as the efficacy of the proposed strategy is still not well documented. In another analysis of the same registry for patients with rheumatoid arthritis (RA) on bDMARDs, not achieving sustained low disease activity or remission had adverse impact on long-term patient function and the accrual of serious adverse events.^14^ However, in SpA there are no studies comparing long-term outcomes (beyond one year of therapy) of patients treated to target compared to those of usual practice.

SpA patients are frequently treated with high-cost drugs, resulting in a significant financial burden on healthcare system.^15^ Using these drugs in patients who will not respond to therapy, besides the significant cost, places patients in potential risks without benefit. Multiple RCTs and registry studies have explored for potential predictors of response to treatment with bDMARDs. Male sex, higher C-reactive protein (CRP) and better function at treatment start were associated with better response to therapy in axial SpA (AxSpA), however with a moderate effect size.^11,16^ Strong –and preferably modifiable – clinical variables at baseline or during therapy that can determine the outcome are not yet known.

Comorbidities in patients with SpA have been shown to have an adverse impact on many patient outcomes, such as functional disability, quality of life, work productivity and mortality.^17–19^ Comorbidities can also affect adverse event incidence during treatment, such as infections. The predictive value of higher comorbidity index on treatment response in SpA patients has not been sufficiently studied, however, recent studies from registries indicate that comorbidities may affect short-term response to therapy with TNFi^20,21^ and more research in this direction is necessary.

Treatment response could also be affected by sociodemographic factors (eg, education, employment), patient lifestyle (exercise, diet, and smoking),^22–24^ and by compliance to therapy. Even though most data come from studies in RA patients, it is shown that there is a high rate of therapy non-compliance^25^ and this could have an adverse impact on long-term prognosis.^26^ EULAR has highlighted the importance of patient education regarding their disease course and management and the effective implementation of lifestyle changes that can improve outcomes, such as exercise and smoking cessation in its recent recommendations.^27–29^

In addition to randomised clinical trials (RCTs), well-structured patient registries are important tools to evaluate treatment effects in “real-world” patients and provide with answers to many types of research questions relevant to clinicians.^30^ Advantages of registries include the diverse population of patients assessed in daily practice, many of which would be excluded from RCTs due to their stringent exclusion criteria (eg, patients with co-morbidities, non-compliance, or specific previous or ongoing co-therapies),^31^ the long follow-up time of monitoring and the possibility to explore for new associations, or predictors of a treatment outcome.

## AIMS OF THE STUDY

The aims of the present study are:

1. To assess the extent of the implementation of T2T strategy in SpA patients of real-world practice, to explore the reasons of possible non-implementation and to evaluate the impact of this therapeutic strategy in the long-term (2 year) prognosis of these patients compared to usual practice.
2. To use validated metrics and collect prospective, accurate, relevant, complete, and up-to-date data of disease parameters, comorbidities and patient lifestyle factors and compliance and evaluate their predictive effect on disease outcomes (safety, function, quality of life, productivity and health indices) in SpA patients starting/switching to a new therapy.

## METHODS

### Study design

This study has a prospective design, and it will take place in the Rheumatology Department of the University Hospital of Heraklion and the University of Crete, Medical School. It has been approved by the Ethics Committee of the University Hospital of Heraklion. Consecutive patients will be enrolled, after their consent, according to the following inclusion criteria: a) adult (≥18 years old) patients with AxSpA or peripheral SpA (perSpA) including psoriatic arthritis (PsA) diagnosed by a rheumatologist, b) regular follow-up at the outpatient department of our hospital or the Rheumatology one-day infusions’ Clinic, and at least 1 visit within the first semester of the study, and c) any prescribed therapy for SpA including NSAIDs, csDMARDs, bDMARDs, or tsDMARDs.

Questionnaires regarding patient characteristics, comorbidities, and disease and treatment characteristics will be collected from patients and physicians at baseline (specific parameters outlined below). After recruitment (months 0 - 6), patients will be prospectively followed-up for 2 years (months 7 – 30) and the implementation of T2T strategy or not (and reasons) will be monitored, along with patient reported outcomes and disease activity scores at specific timepoints (outlined below). Additionally, all adverse events and treatment switches will be recorded at the time they occurred.

A target of at least LDA, based on Ankylosing Spondylitis Disease Activity Score (ASDAS) in AxSpA patients and Disease Activity In Psoriatic Arthritis (DAPSA) index in peripheral SpA and PsA patients will be set for all patients. A follow-up will be considered as compatible with the T2T recommendations if: (a) the patient is re-evaluated within 3 or 6 months according to the guidelines (see below) and (b) disease activity measured using the aforementioned indices is used as a guidance for any necessary treatment modifications. The T2T approach requires adaptation of treatment using the best available therapeutic choices when a patient has not reached the predefined treatment target. In this study, treatment modifications will include increasing the dosage, addition or switching to a new b-/ts-/csDMARD(s), administration of NSAIDs (in AxSpA) and short-course systemic corticosteroids (in PerSpA) or intraarticular steroid injections (in both AxSpA and PerSpA).

### Follow-up intervals and variables collected

As this is a non-interventional study, patient re-assessment intervals will follow the nationally and internationally-issued guidelines, according to which patients with moderate or high disease activity should be re-assessed every (maximum) 3 months, while patients with low disease activity or remission may be re-evaluated every 6 months. From each patient, the following variables will be collected at inclusion visit and/or at regular (3-6-month) follow-up intervals:

- **Patient characteristics**: Socio-demographics (gender, date of birth, education, employment, residence, marital status) and lifestyle (smoking, alcohol, exercise, diet).
- **Disease characteristics**: Date of diagnosis, axial and/or peripheral disease involvement, extraarticular manifestations, SpA classification criteria, imaging (X-Rays and MRI), disease activity and patient function indices (BASDAI, ASDAS,^32^ BASFI,^33^ HAQ,^34^ DAS28, DAPSA^35^) and extraarticular manifestations’ activity indices [psoriasis body surface area (BSA), LEI,^36^ dactylitis count, Inflammatory Bowel Disease flare and uveitis flare (Yes/No)].
- **Treatment characteristics**: Previous treatments (drugs, treatment duration, reason of stop), ongoing drugs (name and dosage of main and concomitant disease treatments, treatment discontinuations and reasons), and compliance to ongoing therapy (MARS-5).^37^
- **Quality of life, productivity and health Indices:** EuroQol 5D (EQ-5D), ASAS-HI,^38^ PSAID,^39^ Hospital Anxiety and Depression Scale (HADS) and Work Productivity and Activity Impairment questionnaire (WPAI).
- **Comorbidities:** All comorbidities, with an emphasis on those included in the Rheumatic Disease Comorbidity Index (RDCI).^40^
- **Adverse events**: All adverse events occurring during follow-up (MedDRA recording system; https://www. meddra.org/faq/meddra-general); grading according to Common Terminology Criteria for Adverse Events (CTCAE) v.5.

Finally, a questionnaire (provided in the **Supplemmentary File**) will be filled by treating physicians after each patient encounter regarding the application of the T2T approach and the reason this might not be applicable or feasible in those patients whose therapy is not intensified despite non achievement of therapy targets.

### Database

Data will be captured using the University of Crete Rheumatology Clinic Registry (UCRCR), a web-based platform developed in collaboration with the Center for eHealth Applications and Services (CeHA) at the Institute of Computer Science, Foundation for Research and Technology – Hellas (ICS-FORTH). UCRCR employs strict security specifications and anonymized patient data, in line with the General Data Protection Regulation (GDPR) and serves as a tool for monitoring patients with immune-mediated systemic diseases of single-center as well as multicenter studies. The database is built to manage a vast array of heterogeneous patient and disease related data and follow-up their changes in time, while it allows for real-time customised exports of data for further analysis.

### Study outcomes

We will assess for the attainment of low disease activity and remission, as well as other treatment outcomes (quality of life, health and function indices, productivity, and adverse events) in patients who were treated or not treated based on the T2T recommendations. As this is not an interventional trial, the length of time over the two years that a patient was followed according to the T2T strategy will be used to classify patients between the T2T and non-T2T strategy. We also plan to evaluate the burden of comorbidities in these patients and its accrual during the study and patient compliance to therapy. Predictors of treatment response will be sought among these and other baseline and early on-treatment variables.

### Statistical analysis

Statistical analysis (months 31–36) will include descriptive results on demographics, proportion of patients with low disease activity and remission, comorbidity indices, compliance, and the implementation of the T2T strategy. Multivariable regression and mixed effects models will be employed to explore predictors of attainment of treatment targets and of long-term (2-year) treatment outcomes among baseline and early on-treatment clinical and demographic characteristics. Due to the phenotypic heterogeneity of SpA, we aim to enroll at least 300 patients, which will allow for statistically robust results to be obtained.

## ANTICIPATED RESULTS AND PROJECT SIGNIFICANCE

Complementary to randomised controlled trials, registry-based studies of real-world patients are important tools to assess long-term effectiveness, safety and pharmacoeconomic data when new therapies and treatment strategies are applied, while they can correlate clinical response with parameters such as comorbidities, co-administered therapies and patient’s compliance. The treat-to-target approach has been found effective in RA and there are indications that it can be effective also in SpA patients, although this is not yet proven.

The significance of the study lies in the following aspects:

1. We will quantify the level of effective control of inflammatory burden in patients with SpA in daily practice.
2. The level of the T2T strategy implementation and the reasons for non-implementation in patients who have not reached treatment targets will be evaluated.
3. The study will assess factors which could possibly be associated with treatment response in SpA, such as several validated disease indices, treatment characteristics and non-commonly collected clinical and patient variables, like comorbidities, sociodemographics and compliance.
4. The predictive value of these factors and the evaluation of the effectiveness of the T2T approach regarding long-term patient outcomes will be explored.

Thus, the successful completion of this study could pave the way for improved and personalised therapy in patients with SpA.

## Notes

**Questionnaire to Physicians concerning Treat-to-Target strategy in SpA**

**Patient’s name**:............................................................ **Date:** ..................

**In present clinical visit, patient has:**

- **Axial SpA**, with **ASDAS**: □ < 1,3 (inactive), □ < 2,1 (low disease activity), □ ≥2.1 (high disease activity)
  *[If* ≤3 months since last treatment switch*,* ***ASDAS improvement****: □ ≥ 1,1 (clinically important) or □ <1,1 ]*
  *If BASDAI is used to monitor patient: □ <4 (LDA/rem) - if 1st trimester on current therapy, BASDAI improved: □ ≥2)*
- **Peripheral SpA** with **DAPSA**: □ < 5 (remission) □ <15 (LDA) □ ≥15 (moderate/high disease activity)
  *[If* ***1st trimester*** *of current therapy,* ***DAPSA improvement****: □ >50% ή □ <50% since therapy start]*
- **Psoriatic arthritis** fulfilling criteria for minimal disease activity (**MDA**) (≥5/7 criteria among:
  □ TJC68≤1 □ SJC66≤ 1 □ BSA<3% □ Enthesitis ≤1 □ VAS global ≤20 □ VAS pain ≤15 □ HAQ ≤ 0.5)

Please circle **one number** that **best represents your decision** in respect to patient’s drug treatment:

**1. Treatment target is achieved**, treatment intensification is not necessary
**2. Intensification** of drug treatment

Drug treatment **is not modified/intensified** despite targets not achieved, due to:

**3. Satisfactory clinical picture**/**largest possible improvement** in a patient with significant articular damage
**4. Disease flare**, probably **transient**, in a patient previously having long-term low disease activity/remission
**5.** Concurrent **fibromyalgia/non-inflammatory pain** which affects disease activity indices
**6. Comorbidities/Fragility**
**7. No alternative drug treatments**
**8. Patient does not wish** treatment intensification
**9. Other reasons** for not modifying/intensifying drug treatment (eg. patient does not receive his treatment properly). Please describe:

**Ερωτηματολόγιο ιατρού για θεραπευτική στρατηγική βάσει προκαθορισμένου στόχου στις ΣπΑ**

**Ονοματεπώνυμο ασθενούς**:............................................................ **Ημερομηνία:** ..................

**Ο ασθενής έχει στην παρούσα επίσκεψη:**

- **Αξονική ΣπΑ**, με **ASDAS** : □ < 1,3 (ύφεση) □ < 2,1 (χαμηλή ενεργότητα), □ ≥2.1 (υψηλή ενεργότητα)
  *[Εάν* ***1<sup>ο</sup> τρίμηνο*** *θεραπείας,* ***βελτίωση ASDAS****: □ ≥ 1,1 (κλινικά σημαντική) ή □ <1,1 ]*
  *Εάν παρακολουθείται με βάση το BASDAI: □ <4 (LDA/ύφεση) και εάν 1<sup>ο</sup> τρίμηνο, βελτίωση BASDAI: □ ≥2)*
- **Περιφερική ΣπΑ** με **DAPSA** □ < 5(ύφεση) □ <15 (χαμηλή ενεργότητα) □ ≥15 (μέτρια/υψηλή ενεργότητα)
  *[Εάν* ***1<sup>ο</sup> τρίμηνο*** *θεραπείας,* ***βελτίωση DAPSA****: □ >50% ή □ <50% από την έναρξη]*
- **Ψωριασική αρθρίτιδα** που πληροί κριτήρια για minimal disease activity (**MDA**) (≥5/7 από:
  □ TJC68≤1 □ SJC66≤ 1 □ BSA<3% □ Ενθεσίτιδα ≤1 □ VAS global ≤20 □ VAS pain ≤15 □ HAQ ≤ 0.5)

**Ποιες ενέργειες θα κάνετε ως προς τη θεραπευτική του αγωγή:**

**3.** έχει επιτευχθεί ο προκαθορισμένος στόχος, δε χρειάζεται εντατικοποίηση της αγωγής
**4. Τροποποίηση/Εντατικοποίηση** της φαρμακευτικής αγωγής

Η εντατικοποίηση της φαρμακευτικής αγωγής **δεν κρίνεται απαραίτητη** λόγω:

**5. Ικανοποιητικής κλινικής εικόνας**/**μέγιστης δυνατής βελτίωσης** ασθενούς με σημαντικές δομικές βλάβες
**6. έξαρσης νόσου**, πιθανότατα **παροδικής**, σε ασθενή σε προηγούμενη χρόνια χαμηλή ενεργότητα νόσου/ύφεση
**7.** Συνυπάρχουσας **ινομυαλγίας**/**πόνου** από **μη φλεγμονώδη αιτία** που επηρεάζει τους δείκτες ενεργότητας νόσου
**8. Συννοσηροτήτων/ευθραυστότητας**
**9. Εξάντλησης εναλλακτικών θεραπειών**
**10. Ο ασθενής δεν επιθυμεί** εντατικοποίηση της αγωγής
**8. Άλλοι λόγοι** που δεν γίνεται εντατικοποίηση της αγωγής (π.χ. ο ασθενής λαμβάνει πλημμελώς την αγωγή του) Παρακαλώ περιγράψτε:
